# Sustainability in pediatric hospitals: An exploration at the intersection of quality improvement and implementation science

**DOI:** 10.3389/frhs.2022.1005802

**Published:** 2022-11-10

**Authors:** Sara Malone, Jason Newland, Sapna R. Kudchadkar, Kim Prewitt, Virginia McKay, Beth Prusaczyk, Enola Proctor, Ross C. Brownson, Douglas A. Luke

**Affiliations:** ^1^Division of Public Health Sciences, Department of Surgery, Washington University in St. Louis School of Medicine, St. Louis, MO, United States; ^2^Pediatric Infectious Diseases, Department of Pediatrics, Washington University in St. Louis School of Medicine, St. Louis, MO, United States; ^3^Department of Anesthesiology and Critical Care Medicine, Pediatrics, and Physical Medicine and Rehabilitation, Johns Hopkins University School of Medicine, Baltimore, MD, United States; ^4^Center for Public Health Systems Science, Brown School, Washington University in St. Louis, St. Louis, MO, United States; ^5^Division of General Medical Sciences, Department of Medicine, Washington University School of Medicine in St. Louis, St. Louis, MO, United States; ^6^Brown School, Washington University in St. Louis, One Brookings Drive, St. Louis, MO, United States; ^7^Prevention Research Center, Brown School, Washington University in St. Louis, One Brookings Drive, St. Louis, MO, United States

**Keywords:** implementation science, clinical sustainability, quality improvement, pediatrics, sustainability capacity

## Abstract

**Background:**

Although new evidence-based practices are frequently implemented in clinical settings, many are not sustained, limiting the intended impact. Within implementation science, there is a gap in understanding sustainability. Pediatric healthcare settings have a robust history of quality improvement (QI), which includes a focus on continuation of change efforts. QI capability and sustainability capacity, therefore, serve as a useful concept for connecting the broader fields of QI and implementation science to provide insights on improving care. This study addresses these gaps in understanding of sustainability in pediatric settings and its relationship to QI.

**Methods:**

This is a cross-sectional observational study conducted within pediatric academic medical centers in the United States. Clinicians surveyed worked with one of three evidence-based clinical programs: perioperative antimicrobial stewardship prescribing, early mobility in the intensive care unit, and massive blood transfusion administration. Participants completed two assessments: (1) the Clinical Sustainability Assessment Tool (CSAT) and (2) a 19-question assessment that included demographics and validation questions, specifically a subset of questions from the *Change Process Capability Questionnaire*, a QI scale. Initial descriptive and bivariate analyses were conducted prior to building mixed-effects models relating perceived QI to clinical sustainability capacity.

**Results:**

A total of 181 individuals from three different programs and 30 sites were included in the final analyses. QI capability scores were assessed as a single construct (5-point Likert scale), with an average response of 4.16 (higher scores indicate greater QI capability). The overall CSAT score (7-point Likert scale) was the highest for massive transfusion programs (5.51, SD = 0.91), followed by early mobility (5.25, SD = 0.92) and perioperative antibiotic prescribing (4.91, SD = 1.07). Mixed-effects modeling illustrated that after controlling for person and setting level variables, higher perceptions of QI capabilities were significantly related to overall clinical sustainability.

**Conclusion:**

Organizations and programs with higher QI capabilities had a higher sustainability capacity, even when controlling for differences at the individual and intervention levels. Organizational factors that enable evidence-based interventions should be further studied, especially as they relate to sustainability. Issues to be considered by practitioners when planning for sustainability include bedside provider perceptions, intervention achievability, frequency of delivery, and organizational influences.

## Introduction

### Implementation science and sustainability capacity

While recent implementation science work has focused on improving how programs and interventions get initially implemented within complex settings, the impact of an evidence-based intervention is not fully realized without appropriate sustainment over time. Studies have consistently shown that fewer than half of practice changes are sustained, with one review finding only 4% of practices in healthcare reporting sustainment (1–3).

Sustainability has been defined as “the extent to which an evidence-based intervention can deliver its intended benefits over an extended period of time after external support… is terminated” (4). While research on sustainability is increasing, it is still relatively poorly understood (5–7). One important research opportunity is identifying the determinants of sustainment of evidence-based interventions (8, 9). Some examples of relevant determinants include individual interested parties, multi-professional relationships, and organizational culture (10–13). While there has been work initially conceptualizing some of these determinants as sustainability capacity, there is still much to be done before we can understand all the factors that influence sustainability. To understand how to intervene to ensure sustainment of evidence-based practices, it is crucial to advance the study of sustainability determinants and theory in clinical settings.

### The relevance of quality improvement for studying clinical sustainability

Health care systems have developed with an emphasis on continual improvement, resulting in numerous theories and methods being developed and refined (14–19) focusing on how healthcare delivery can be improved, resulting in better patient safety and more positive health outcomes. While there are different histories and approaches to improvement, quality improvement and implementation science are aligned in their focus on improving care delivery and outcomes. *Quality improvement (QI)* is focused on identifying local, context-specific problems and rapid correction. While having a scientific and theoretical basis, QI is a more applied science within the hospital system (20). There are opportunities to improve our understanding of implementation science in clinical settings by bringing in QI science.

QI is aimed at realizing improvement within specific metrics, which makes it helpful in project management and execution in busy and under-resourced settings. Some QI studies have demonstrated an ability to sustain their practices (21, 22). However, other literature has cited difficulties with sustaining changes (23). Some research has begun to target determinants of sustainment of practice change (24).

*Implementation science*, which also focuses on improving healthcare services, according to Mittman, “generally seeks to develop and rigorously evaluate fixed implementation strategies to address implementation gaps across multiple sites” (25). This has created a dichotomy where implementation scientists focus on information that can be scaled and generalized, while QI work has aimed its interventions at individual needs and corrections. However, the fields overlap, with their common focus on improving the delivery of evidence-based practices to benefit patients.

### The importance of context in clinical sustainability

One important class of determinants of sustainability are characteristics of the *context* within which the intervention is carried out. Context has been defined by May et al. as “the physical, organizational, institutional, and legislative structures that enable and constrain…people and procedures” (26). It follows, then, that understanding outcomes requires knowledge of the environmental context within which the system is embedded (e.g., staffing, organizational climate) (27). Since these contexts vary by setting, there is a need for “unpacking” these contextual factors within clinical care to enumerate key contextual variables, prioritize those most salient, and measure these variables across settings (28, 29).

The clinical healthcare environment consists of unique provider dynamics, workflow challenges, and complexities to overcome when evaluating sustainment of practices over time (30). Clinical care is best understood through practices and procedures that occur, relying heavily on frontline providers who are conducting activities highly integrated with the rest of the workflow. The time horizon for implementation and impact is often shorter in clinical sustainability than in public health, allowing patient and system-level changes to be seen more immediately by those providing care. To understand these differences, clinical sustainability must be distinguished from sustainability more broadly. Clinical sustainability has been defined as “the ability of an organization to maintain structured clinical care practices over time and to evolve and adapt these practices in response to new information (31).”

The workflows, team composition, and relationship to patients and families are some of the factors that make pediatrics a unique care delivery setting. For example, children's hospitals require multidisciplinary expertise focused on the experiences of childhood. While medical specialists have different training for pediatrics, there are also different professional roles regularly involved in pediatric settings, including clinical social work and child life specialists. Additionally, pediatric hospitals must focus on the parents and caregivers, whereas adult settings are less concerned about caregivers and less frequently have individuals other than the patient providing consent for treatment (32). The research base for children is more limited due to ethical and practical issues with recruitment and testing (33). Providers often express concern with the available evidence due to origination in adults and concerns about the imperfect translation of evidence to pediatric settings (34). All these differences require special attention to be paid to pediatric health settings.

### Goals and research questions

This study addresses some of these gaps in understanding sustainability in pediatric settings and its relationship to QI. More specifically, this study assesses different individual and intervention characteristics, including quality improvement capabilities, and their association with clinical sustainability capacity. By examining the ability of a healthcare organization to implement and continue to deliver high-quality care, the study aims to answer the following questions:

(1) What specific individual and organizational factors are related to clinical sustainability capacity?
(2) How does quality improvement capability correspond to sustainability of clinical programs?

Results from this study will help us to understand if there are any individual, intervention, or quality improvement determinants that contribute to sustainability capacity and could point toward future areas of intervention. This will help advance the science of sustainability through the development of links between determinants and sustainability capacity.

## Methods

This is a cross-sectional observational study conducted within pediatric academic medical centers in the United States. The study included healthcare professionals affiliated with one of three evidence-based clinical programs and uses multilevel modeling to assess hospital-level contextual factors and their associations with sustainability capacity.

### Settings

Three multicenter national hospital clinical programs were included in this study. Thirty sites participated in the study amongst the three programs. All thirty sites were engaged in evidence-based practice change that involve multi-professional teams. These are all programs that emphasize delivering evidence-based interventions in different units and teams. While some sites had multiple programs that were eligible for the study, they were treated as separate sites due to the unique resources and personnel in each clincial unit. Each site had been delivering the program for a different length of time.

The three evidence-based interventions that the sites were focused on were: antibiotic prescribing in clean/clean-contaminated surgeries (35), early mobility within the pediatric intensive care unit (36), and massive transfusion blood administration (36, 37). These are all internationally recognized guidelines and evidence-based practices (38–40). Table 1 outlines each intervention and the multi-professional team involved. For ease of describing the practices, the surgical antibiotic practice will be referred to as an antimicrobial stewardship program (ASP) throughout.

**Table 1:** Description of three pediatric interventions.

	**ASP^a^**	**Early mobility**	**Massive transfusion**
Description	Appropriate antibiotic prescribing practices in clean and clean-contaminated surgical cases	A care bundle focused on reduction of delirium and sedation to begin early rehab for children that are critically ill	Practices that allow for rapid distribution and administration of blood product
Professions	Pharmacist, physician, physician assistant, nurse practitioners	Nurse, physician, respiratory therapy, physical therapy, occupational therapy	Physician, pharmacist, blood banker
Disciplines	Infectious disease, surgery	Critical care	Emergency medicine, blood bank, intensive care

a Antimicrobial stewardship program (ASP) perioperative antibiotic prescribing program.

### Participants and recruitment

Data were collected during October 2020—July 2021 from 181 multi-professional clinicians involved in the pediatric evidence-based practices described above. A group of institutions participating in delivery of these interventions was generated through collaboration amongst the study team. National program leads were used to identify team leads at each site for each of the three practices, with a total of 40 sites originally identified. Site leads were then contacted and asked about their site participation. If team leads agreed, a list of site participants was provided to the study team, which was defined as any individual involved in the relevant clinical care practice in their setting. These individuals were then recruited to complete the survey over email and were invited to forward the email to anyone else in their organization that participated in delivery of the intervention. Known participants were contacted twice *via* email and asked to participate in an online survey that was conducted using Qualtrics (Qualtrics, Provo, UT). Overall, 30 sites participated in the study (Table 1). All participating sites were US based academic medical centers with either (1) dedicated pediatrics care or (2) a freestanding children's hospital. From these sites, 181 individuals responded to the survey. There were no incentives provided for participating in the study. The study protocol was reviewed and approved by Washington University Human Research Protection Office (202102017).

### Data sources

The survey instruments were:

- Clinical Sustainability Assessment Tool (CSAT) (41)—This measure assesses clinical sustainability capacity and includes seven domains: engaged stakeholders, engaged staff and leadership, organizational readiness, monitoring and evaluation, implementation and training, outcomes and effectiveness, and workflow integration. There are 35 questions, all completed on a seven-point Likert scale with options ranging from: not at all—to a great extent. There is also a “not able to assess” option for each question. This instrument has demonstrated reliability and is one of the few instruments developed to assess sustainability in clinical settings (42).
- *Validation questions*—This is an additional set of questions that gathers information about the nature of the evidence-based intervention as well as other organization characteristics that assist in understanding the validity of the CSAT. The questions were grouped into two categories: questions about the organization and those about the intervention. A subset of the organization questions were taken from the Change Process Capability Questionnaire, a QI assessment utilized by the Agency for Healthcare Research and Quality (AHRQ) (43). Additional organizational, intervention, and individual questions are described below.
- Demographic questions—A set of questions provided information about the individual taking the assessment. These include the role, profession, and the environment within which the individual usually practices (e.g., adult vs. pediatrics, inpatient vs. outpatient).

The full instruments can be found in Supplementary material A and B.

### Variables, data management, and analysis

The variables of interest for this project can be found listed in Table 2. In addition to data collected to understand quality improvement capability, other data were collected to assess organizational and individual determinants that could influence the sustainability of pediatric clinical programs. This is further explained below within the description of mixed-effects modeling.

**Table 2:** Variables included in study.

**Variable**	**Variable type**	**Source**
**Dependent variables**	
Sustainability capacity [CSAT]	Continuous [averaged across 7 domains]	Clinical sustainability assessment tool
**Independent variables**	
Quality improvement capability^a^	Continuous [average of 6 questions]	Validation survey
**Covariates: individual**	
Role	Categorical	Demographics
Profession	Categorical	Demographics
Position	Categorical	Demographics
Service environment	Categorical	Demographics
**Covariates: organizational**	
Organization type	Categorical	Demographics
Size	Ordinal [3 levels]	Demographics
Urban/Rural	Categorical	Demographics
**Covariates: intervention**	
Length of practice	Ordinal [5 levels]	Validation survey
Strength of evidence	Ordinal [5 levels]	Validation survey
Importance	Ordinal [5 levels]	Validation survey
Achievability	Ordinal [5 levels]	Validation survey
Frequency of delivery	Ordinal [5 levels]	Validation survey

a This is a calculated score, comprised of five items from the Change Process Capability Questionnaire (45).

#### Sustainability capacity

Sustainability capacity was the main dependent variable for this study. Capacity was represented as the CSAT score for each domain as well as an overall sustainability capacity score. The seven domain scores were calculated as a simple average of the five items within each subscale. Scores can range from 1 to 7, where a higher score indicates a higher sustainability capacity. The total CSAT score was calculated as an average of the seven domain scores, again ranging from 1–7. This total score represents the perceived sustainability capacity for the specific clinical setting, where higher numbers indicate a greater capacity.

#### Quality improvement capability

Quality improvement capability was the main independent variable for this study. Six questions were chosen from the Change Process Capability Questionnaire as a proxy for QI work conducted at the site level (43). This score reflected the overall site relationship to QI and use of QI strategies. Scores could range from 1 to 5, where higher scores indicated a higher extent of quality improvement capabilities within their setting. All six questions included in the quality improvement capability construct were assessed individually and as a scale. One of the six items was re-coded, as it was initially reverse coded.

One item performed poorly during reliability testing, indicating it was not measuring the same latent construct of QI capability. This item was ultimately removed to create a 5-item scale of quality improvement capability. This included history of use of QI methods, assessment of QI culture, and strategies that were used in the setting. This scale was utilized in the rest of the study as an average. The value for Cronbach's alpha for the construct was α = 0.83, indicating very good reliability (44).

#### Other covariates

Other covariates of interest were assessed at the individual, intervention, and organization level.

Participants reported three organizational characteristics: the type of organization, staff size, and location. Organizational variables were assessed for distribution and some responses were collapsed. Environment was re-coded to a binary variable, assessing those who worked at primarily at an academic medical center compared to those who also deliver care in other settings, such as community hospitals or urgent care. All individuals identified their organization as located in an urban area with many employees, so these two variables were eliminated from further analyses.

Individuals were asked to assess their perception of the intervention in five different ways. First, people reported the length of time, in years, that they believed the intervention had been implemented in their setting. Next, they were asked to identify the strength of evidence supporting the intervention or practice (5 options, from very weak to very strong). Third, participants reported their perception of how important the intervention was to provide quality care within their setting (5 options, from not at all important to very important). Participants also assessed their perception of how easy the practice was to implement within the setting, described as achievability (5 options, from very difficult to very easy). Finally, they were asked about the frequency of delivery, or how often those in their care received the intervention (from not at all to all the time).

Four questions were asked to understand characteristics about the participants. All four individual-level variables were assessed and three were re-coded to assist with distribution across the data. The participant role remained a categorical variable as collected, with individuals reflecting all types of involvement in the implementation team. The setting was recoded to a binary variable, with individuals identified as those practicing in one setting vs. more than one setting (inpatient *and* outpatient). Position was recoded due to the frequency of bedside clinicians included in the sample, and the other three positions of leadership, administration, and research were collapsed into a single response category. Finally, the individual profession was collapsed into nurses, physicians, and all others (i.e., respiratory therapy, physical therapy, social work, and pharmacists).

### Data analysis

The data were recoded, cleaned, and analyzed in R. Both the CSAT scores and a Quality Improvement Capability Score were calculated, derived from the questions taken from the AHRQ Change Process Capability Questionnaire (45). The data were analyzed in three phases. First, descriptive statistics were generated to assess each individual variable as well as begin to understand sustainability across the programs. Next, bivariate statistical analysis was conducted to understand the relationship between some of these variables and sustainability. Finally, multi-level models were built to answer questions about the relationship of quality improvement capability to clinical sustainability capacity.

A multilevel analysis was conducted to identify associations of individual-level and contextual factors with clinical sustainability capacity. A two-level multilevel structure was utilized, where healthcare staff was nested with clinical care sites. Using multilevel analysis helped address clustering and account for contextual information at the organizational level (46).

The multilevel modeling equation for this two-level structure was:


$$\begin{array}{l} Level\;1:\;Sustain_{ij}=\;\beta_{0j}+\beta_{1j}QIC_{ij}+{\beta_{2j}Individual_{ij}+r}_{ij}\\ Level\;2:\;\beta_{0j}=\;\gamma_{00}+\gamma_{01}Org_{j}+u_{0j}\\ \beta_{1j}=\;\gamma_{10}+\gamma_{11}Org_{j}+u_{1j}\\ \beta_{2j}=\;\gamma_{20}+\gamma_{21}Org_{j}+u_{2j} \end{array}$$

In this equation, level one represented the participant level differences in their sustainability capacity score. The second-level represents the differences at the organization or site level. *Th*e dependent variable of interest is sustainability capacity (Sustain). Sustainability capacity was modeled as a function of quality improvement capability scores measured at the person-level (*QIC*) and other person-level covariates (Individual). The covariates at the second-level variable, Org, included the program type and perceptions about the interventions evidence, achievability, and frequency of delivery.

This allowed for a model that can answer one of the main questions of interest requiring a multi-level model, which is how perceived quality improvement capabilities predicts sustainability after controlling for other individual and intervention level characteristics. This model also assisted in answering questions about other relevant determinants of sustainability capacity. This model was built in a block fashion, with intermediate models produced before the final model focusing on the role of quality improvement capability. This block model-building approach allows us to examine the role of QI capability on sustainability after controlling for the other individual and site-level covariates.

The models were built sequentially, starting with a null model to test ICC and then adding level one and level two variables in sequentially to subsequent models. Finally, the QI capability score was added, forming the final model.

## Results

### Describing sustainability across programs

#### Participant and setting descriptive statistics

A total of 181 individuals from three different programs and 30 sites were included in the final analysis. Individual demographics of interest are included in Table 3. Individuals most frequently worked in a single practice setting (e.g., inpatient) (74%) and were involved in direct patient care (70%). About half of the participants were physicians (48%), although all professions were recruited to participate within each setting.

**Table 3:** Participant characteristics and clinical role.

	**ASP^a^**	**Early**	**Massive**
		**mobility**	**transfusion**
Total # of sites in sample	10	8	12
Total # of people in sample	53	88	40
**Setting**			
Single setting	34	80	20
Two + settings	18	8	20
**Profession**			
Physician	34	28	26
Nurse	1	26	6
Other	18	34	8
**Position**			
Direct patient care	32	77	18
Other	21	11	22

a Antimicrobial stewardship program (ASP) perioperative antibiotic prescribing program.

Individuals within the study primarily identified their practice group as pediatrics across all three programs. Individuals reported their practice environment largely as academic medical centers (84%). Most people described the intervention as existing at their site for <5 years and believed the evidence for the intervention to be strong, with a mean score of 4.22 (SD = 0.74). Participants demonstrated bimodal reporting for importance, reporting the intervention to be either very unimportant or important. Those participating in ASP (M = 3.20, SD = 0.74) and early mobility (M = 3.08, SD = 0.69) reported individuals receiving the intervention more frequently than those in massive transfusion programs (M = 2.77, SD = 0.96). Table 4 presents the intervention level descriptive statistics.

**Table 4:** Intervention level descriptive statistics by program.

	**Program**		
	**ASP^a^**	**Early**	**Massive**
		**mobility**	**transfusion**
**Length of practice**			
Less than 1 year	4	5	0
1–5 years	4	73	14
6–10 years	15	1	13
>10 years	7	1	10
**Strength of evidence**			
Very weak	0	0	0
Weak	1	0	2
Neither weak nor strong	8	6	8
Strong	29	33	15
Very strong	0	0	0
**Importance of intervention**			
Very unimportant	5	12	0
Somewhat unimportant	0	0	0
Neither important or unimportant	0	0	0
Somewhat important	9	9	4
Important	40	67	36
**Achievability of implementation**			
Very difficult	0	0	1
Somewhat difficult	27	18	7
Neither easy nor difficult	6	20	12
Somewhat easy	12	29	11
Very easy	0	3	2
**Frequency of delivery**			
None of the time	1	0	0
Some of the time	7	18	23
Most of the time	24	45	2
All of the time	19	25	14

a Antimicrobial stewardship program (ASP) perioperative antibiotic prescribing program.

#### CSAT scores

Table 5 presents the subscale and overall CSAT scores in total and by each program. The overall CSAT was highest for massive transfusion programs (5.51). Each program had different high-performing domains. The standard deviation highlights variability within each of the scores.

**Table 5:** CSAT subscale and total score by program.

**CSAT**	**ASP^a^**	**Early mobility**	**Massive transfusion**	**Total across**
**Subscales**	**(*n* = 53)**	**(*n* = 88)**	**(*n* = 40)**	**programs**
Engaged staff and leadership	5.21 (1.29)	5.41 (1.14)	5.71 (1.08)	5.43 (1.18)
Engaged stakeholders	4.62 (1.19)	5.56 (1.02)	5.26 (1.31)	5.22 (1.20)
Organizational readiness	5.13 (1.17)	4.98 (1.17)	5.65 (0.97)	5.40 (1.15)
Workflow integration	5.01 (1.17)	5.38 (1.04)	5.6 (0.98)	5.40 (1.09)
Implementation and training	4.53 (1.46)	4.84 (1.31)	5.33 (1.22)	5.00 (1.36)
Monitoring and evaluation	4.64 (1.58)	4.68 (1.52)	5.31 (1.55)	5.00 (1.56)
Outcomes and effectiveness	5.46 (1.21)	5.93 (0.89)	5.60 (0.91)	6.00 (1.02)
Total CSAT score	4.91 (1.07)	5.25 (0.92)	5.51 (0.91)	5.20 (0.98)

a Antimicrobial stewardship program (ASP) perioperative antibiotic prescribing program.

Cells contain averages and standard deviations.

Overall, the scores indicate there was variation by program across each of the domains. Transfusion programs had higher scores in five of the domains, with the mobility programs having the highest domain averages in the other two.

#### Quality improvement capability scores

The quality improvement capability scores were calculated using the average of the five items that were included after calculating Cronbach's alpha. Table 6 presents the item and scale averages and standard deviation for each practice as well as across the three programs. The lowest item mean was for QI in the past year and the highest was for using QI skills. The ASP and massive transfusion programs had the highest overall QI capability scores, with ASP being slightly higher. Like with CSAT scores, the standard deviation indicates there was variability within the QI capability scores.

**Table 6:** Quality improvement items from Change Process Capability Questionnaire.

**Question**	**ASP^a^**	**Early mobility**	**Massive transfusion**	**Total**
Our clinical team understands and uses quality improvement skills effectively.	4.43 (0.69)	4.18 (0.77)	4.35 (0.80)	4.29 (0.76)
Our clinical team has changed or created systems in the organization that make it easier to provide high quality care.	4.40 (0.69)	4.16 (0.83)	4.33 (0.83)	4.27 (0.79)
We choose new processes of care that are more advantageous than the old to everyone involved (patients, clinicians, and our entire clinical team).	4.06 (0.79)	3.93 (0.80)	4.23 (0.86)	4.03 (0.82)
The working environment in our clinical team is collaborative and cohesive, with shared sense of purpose, cooperation, and willingness to contribute to the common good.	4.25 (0.87)	4.13 (0.84)	4.30 (0.76)	4.20 (0.83)
Our clinical team has greatly improved quality of care in the past year.	4.11 (0.75)	3.91 (0.79)	4.00 (0.75)	3.99 (0.77)
Total score	4.25 (0.55)	4.06 (0.61)	4.24 (0.67)	4.16 (0.61)

a Antimicrobial stewardship program (ASP) perioperative antibiotic prescribing program.

### Association of quality improvement capability and clinical sustainability

**Figure 1:**
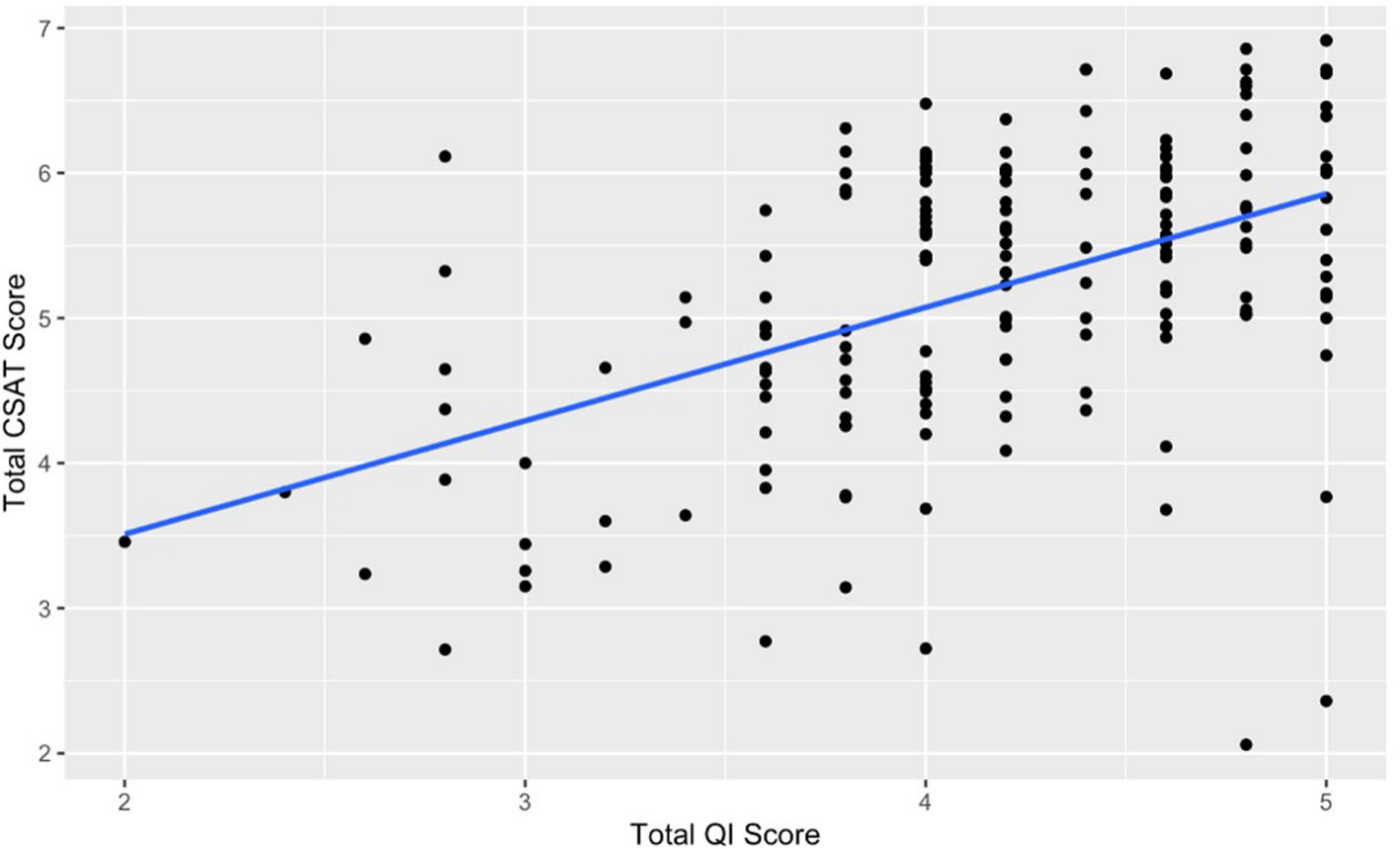
Relationship between total CSAT score and total QI score.

Figure 1 shows the relationship between total QI capability score and total CSAT scores. There is a moderately strong, positive association between these two variables (r = 0.49, *p* < 0.001). This relationship illustrates that an increase in QI capability is associated with higher CSAT scores.

### Model relationships between individual, site-level, and quality improvement covariates with clinical sustainability

After assessing both univariate and bivariate statistics, multilevel mixed-effects modeling was conducted. The models are summarized in Table 7. Four models are presented, starting with a null model (no covariates), an initial substantive model with individual-level covariates, a multilevel model with both individual and site-level covariates, and then a final model with QI capability scores.

**Table 7:** Sustainability capacity modeled with individual, intervention, and organizational predictors.

	**Null model**		**Level 1 (person) variables**			**Level 2 (setting) variables**			**Final model with QI**		
	**Coef**.	**95% CI**	**Coef**.	**(95% CI)**	***p*** **-value**	**Coef**.	**95% CI**	***p*** **-value**	**Coef**.	**(95% CI)**	***p*** **-value**
Intercept	5.15	(4.95, 5.35)	4.91	(4.43, 5.40)		1.25	(0.07, 2.46)		−0.29	(−1.44, 0.86)	
**Setting (reference: single)**												
Setting: multiple			0.12	(−0.23, 0.47)	0.50	0.04	(−0.27, 0.35)	0.80	0.07	(−0.21, 0.36)	0.64
**Position (reference: bedside)**										
Position: other			**0.40**	**(0.04, 0.76)**	**0.03**	0.19	(−0.13, 0.52)	0.28	0.18	(−0.11, 0.48)	0.26
**Profession (reference: nurse)**										
Profession: physician			0.09	(−0.35, 0.52)	0.70	0.30	(−0.09, 0.71)	0.16	0.24	(−0.11, 0.60)	0.22
Profession: other			0.15	(−0.28, 0.59)	0.51	0.36	(−0.03, 0.78)	0.10	0.36	(0.02, 0.73)	0.07
**Role (reference: team leader)**										
Role: administration			0.05	(−0.52, 0.63)	0.87	0.13	(−0.38, 0.65)	0.64	0.19	(−0.27, 0.66)	0.44
Role: participating			−0.07	(−0.45, 0.32)	0.73	0.00	(−0.34, 0.34)	0.98	−0.05	(−0.35, 0.26)	0.75
Role: evaluator			−0.46	(−0.45, 0.31)	0.30	−0.45	(−1.16, 0.30)	0.26	−0.37	(−1.00, 0.31)	0.30
Role: clinical staff			−0.04	(−1.30, 0.39)	0.84	−0.12	(−0.47, 0.22)	0.50	−0.09	(−0.39, 0.23)	0.60
**Program (reference: ASP)**										
Program: early mobility						0.33	(−0.09, 0.74)	0.16	0.37	(0.03, 0.70)	0.06
Program: massive transfusion						**0.64**	**(0.23, 1.06)**	**0.01**	**0.65**	**(0.31, 0.99)**	**0.002**
**Environment (reference: academic)**										
Environment: other						0.14	(−0.25, 0.66)	0.50	0.11	(−0.21, 0.50)	0.55
Int. Importance (reference: not important)										
Int. importance: important						−0.21	(−0.78, 0.33)	0.48	−0.28	(−0.80, 0.20)	0.29
Int. importance: very important						−0.29	(−0.75, 0.15)	0.23	−0.35	(−0.76, 0.04)	0.10
Intervention: strength of evidence						**0.45**	**(0.26, 0.65)**	**< 0.001**	**0.40**	**(0.24, 0.58)**	**< 0.001**
Intervention: length of implementation						0.05	(−0.13, 0.20)	0.54	−0.05	(−0.21, 0.07)	0.43
Intervention: achievability						0.15	(−0.00, 0.31)	0.07	0.10	(−0.03, 0.24)	0.17
Intervention: frequency of delivery						**0.34**	**(0.17, 0.51)**	**< 0.001**	**0.21**	**(0.05, 0.36)**	**0.02**
**Quality improvement capability**									**0.65**	**(0.46, 0.85)**	**< 0.001**
Model fit			**AIC**	509.7		**AIC**	471.0		**AIC**	434.1	
Model improvement (LR Chi-squared)					56.7 (*p* < 0.01)		38.9 (*p* < 0.01)	

Bolded parameters indicate significance at p < 0.05.

#### Null model

The ICC calculated from the null model was 0.12, indicating some variability that is accounted for by the different sites. This non-zero value supports the approach of using mixed-effects modeling to account for clustering of individual-level scores within the specific sites (46).

#### Model with level 1 variables

All level one variables were added to the model at the same time. While level one variables enhanced the model, only one was a significant predictor of sustainability scores within the three programs. Individuals who identified as being primarily in positions other than bedside providers perceived higher sustainability capacity (Coef. = 0.40, *p* < 0.05).

#### Model with level 2 variables

Level two variables were added in two phases to the model. First, organizational variables of program and environment were added. Subsequently, the intervention characteristics were added. The AIC values decreased with the addition of these variables and lower AIC values indicate better fit. The transfusion program staff reported higher CSAT scores relative to the ASP programs (Coef. = 0.64, *p* < 0.05). Higher perception of strength of evidence for a program also resulted in higher CSAT scores (Coef. = 0.45, *p* < 0.05). Individuals that reported higher frequency of delivery, meaning the intervention was delivered more frequently, also reported higher overall CSAT scores (Coef. = 0.34, *p* < 0.05). The perceived ease of implementation and length of time in practice were not significant.

#### Final model

Finally, the five-item quality improvement capability construct was added to the overall model. The AIC decrease suggests that the model was improved through the addition of this construct. The quality improvement capability variable was also significant (Coef. = 0.65, *p* < 0.05). In this model, intervention frequency, the strength of evidence, and transfusion program remained significant. This final model is a significant improvement over the level-2 model (LR Chi-square = 38.9, *p* < 0.01).

### How quality improvement influences sustainability capacity

After the final model was completed and assessed, further analyses were conducted to understand more about the direction and strength of the relationship between quality improvement capability score and CSAT total score. To understand how quality was operating through the sustainability score, the entire model was run with each of the seven CSAT subscale scores as the dependent variable. The final model with all covariates was run, and the parameter estimates for the QI variable for each of the seven models are presented in Table 8. Quality improvement capabilities were positively and significantly associated with CSAT subscale scores for every domain. Quality improvement capability functioned most strongly through monitoring and evaluation and organizational readiness and least through engaged stakeholders and outcomes and effectiveness. However, an increase in quality improvement capability scores led to a significant increase in CSAT domain scores in all the seven domains.

**Table 8:** Mixed effect models for each subdomain, focused on QI variable.

**CSAT domain**	**Quality improvement variable**	
	**in final model**	
	**Coef**.	**SE**
Engaged stakeholders	0.38	0.16
Outcomes and effectiveness	0.43	0.13
Engaged staff and leadership	0.49	0.15
Workflow integration	0.64	0.14
Implementation and training	0.70	0.17
Organizational readiness	0.87	0.13
Monitoring and evaluation	0.93	0.19

All parameters were significant at p < 0.05.

## Discussion

### Sustainability and quality in pediatric hospital care

The construct of QI capability is especially important in pediatric hospital care due to its extensive engagement with the field of quality (47). This study assessed the relationship of various individual and organizational constructs to sustainability capacity. These results show that after controlling for the person and setting level variables, perceptions of higher QI capabilities are significantly related to overall clinical sustainability scores. Our research suggests that QI capability within the hospital is related to the capacity to sustain evidence-based practices after implementation, highlighting a way to consider the relationship of QI theory with implementation science.

The measure of quality improvement capability within the hospital was found to be related to overall sustainability capacity. This study responds to foundational calls within the field of sustainability. Additionally, this study highlights quality improvement processes within healthcare that can serve as a bridging factor, or enabling condition, between larger health delivery organizations and individual high-performing healthcare delivery teams (48, 49). Future work should focus on the systems that facilitate or hinder both QI and sustainability. While this study offers information related to how these constructs are measured in pediatric hospital settings, this research is limited by the sample size and only provides data focused in a single practice environment. Given that other types of programs and practices certainly have different characteristics within the hospital and in other settings, there would be benefit to conducting a larger study both in pediatrics and in other contexts.

Various other factors related to the intervention were significantly related to higher sustainability capacity. Implementors should focus on how different clinicians assess the quality of evidence during implementation and sustainment. The frequency of delivery was consistent with anticipated delivery of these different interventions in routine care. Results highlighting the frequency of delivery creating more capacity for sustainability could potentially function through the domain of workflow integration and is supported by other literature highlighting the importance of routinization into the workflow (50, 51). Future research ought to consider how to sustain interventions in relationship to intervention differences (i.e., acuity, frequency, etc.) (52).

#### Implications for healthcare delivery

This study has implications for implementation practice. First, the CSAT should be considered as a useful tool during QI and/or implementation efforts. Second, this relationship between QI and implementation provides insight into strategies and methodologies that should be considered for training and implementation.

Our findings rely on the use of the Clinical Sustainability Assessment tool. The CSAT scores were consistent, regardless of individual-level characteristics. This study reinforces that the CSAT is a pragmatic tool that can be used by clinicians for evaluation and planning to sustain programs and practices. Additionally, this understanding of how QI initiatives bolster sustainability indicates that utilizing QI methodologies should be considered with planning strategies for implementation efforts.

### Implementation science and quality improvement

This study responds to a theoretical question that has been posed within improvement sciences about the relationship between implementation science and QI. Easterling et al. found implementation science and QI literature to be separate bodies of work when they were assessing learning health system literature (49). QI has been described as an applied science that provides tools and theories to assist in rapid improvement at a local level (53) while implementation science has focused more broadly on the processes for change, context alignment, and outcomes related to both implementation as well as patient health (54–56).

Sustainability may be better understood and enhanced by more closely linking QI and implementation science to provide insights on how to improve care delivery. This study highlights how using theories and tools from both QI and implementation science can enhance our understanding of how to best ensure sustainability of our efforts to improve healthcare quality. Specifically, drawing from these two fields allows for a better understanding of the needs to assess impact to the practitioner (QI), system level care outcomes, as well as the integration into the practice environment and process of implementing change (implementation science). To be successful, research on sustainability determinants in healthcare must address the existence of QI as a relevant influence in the field. This research responds to calls to advance research on sustainability and sustainment (8, 30), and future studies should be focused on organization and intervention level determinants of sustainability as well as their sustainment.

### Limitations

This study draws it strength from being a survey of frontline clinicians engaged in the delivery of these programs. A combination of recruitment strategies was utilized, resulting in an inability to track overall response rate and understand a potential selection bias for those who self-selected to complete the assessment. This survey also reports individual perceptions of these constructs, which are subjective measures. Future research should focus on objective measurement of these constructs and outcomes. By using perception of these constructs, we can assess how clinicians understand the intervention in their clinical environment, which is relevant and can highlight differences in understanding practice delivery within a single setting.

## Conclusion

This study sought to understand the influence of QI on sustainability in pediatric healthcare settings. We found that sustainability capacity is influenced by the following: the perception of evidence, individual roles, frequency of delivery, and QI capabilities of the setting. This is one of these first studies to show a strong relationship between QI and intervention sustainability. This work helps bring together theory and research from QI science and implementation science. By doing this, we highlight the opportunity to improve healthcare delivery by integrating these relevant fields of study.

## Data availability statement

The raw data supporting the conclusions of this article will be made available by the authors, without undue reservation.

## Ethics statement

The studies involving human participants were reviewed and approved by Washington University in St. Louis, HRPO. Written informed consent for participation was not required for this study in accordance with the national legislation and the institutional requirements.

## Author contributions

SM led the process of designing, collecting data, and the writing of the manuscript. JN and SK both assisted with the design of the study, facilitated data collection, and edited the manuscript. VM and KP assisted with data collection, study design, and edited the manuscript. BP, EP, and RB assisted with planning the study, supervision of the project, and drafting of the manuscript. DL assisted with planning the study, data analysis, supervision of the project, and drafting of the manuscript. All authors reviewed and approved the final manuscript.

## Funding

This study was supported in part by the National Institute of Aging K01AG071749 (BP). This study was supported in part by the National Cancer Institute (P50CA244431), the National Institute of Diabetes, and Digestive and Kidney Diseases (P30DK092950 and P30DK056341), the Centers for Disease Control and Prevention (U48DP006395), and the Foundation for Barnes-Jewish Hospital. This study was supported in part by AHRQ R01HS026742-03 (Newland, Malone). The findings and conclusions in this paper are those of the authors and do not necessarily represent the official positions of the National Institutes of Health or the Centers for Disease Control and Prevention.

## Conflict of interest

The authors declare that the research was conducted in the absence of any commercial or financial relationships that could be construed as a potential conflict of interest.

## Publisher's note

All claims expressed in this article are solely those of the authors and do not necessarily represent those of their affiliated organizations, or those of the publisher, the editors and the reviewers. Any product that may be evaluated in this article, or claim that may be made by its manufacturer, is not guaranteed or endorsed by the publisher.

## References

[1] Scheirer M. Is Sustainability possible? *a* review and commentary on empirical studies of program sustainability. Am J Eval. (2005) 26:28. 10.1177/1098214005278752

[2] Wiltsey Stirman S Kimberly J Cook N Calloway A Castro F Charns M. The sustainability of new programs and innovations: a review of the empirical literature and recommendations for future research. Implement Sci. (2012) 7:17. 10.1186/1748-5908-7-17 22417162 PMC3317864

[3] Klaic M Kapp S Hudson P Chapman W Denehy L Story D. Implementability of healthcare interventions: an overview of reviews and development of a conceptual framework. Implement Sci. (2022) 17:10. 10.1186/s13012-021-01171-7 35086538 PMC8793098

[4] Rabin BA BR. Terminology for dissemination and implementation research Dissemination and implementation research in health: translating science to practice. (2017). p. 19–45.

[5] Kaplan HC Provost LP Froehle CM Margolis PA. The Model for Understanding Success in Quality (MUSIQ): building a theory of context in healthcare quality improvement. BMJ Qual Saf. (2012) 21:13–20. 10.1136/bmjqs-2011-000010 21835762

[6] Breimaier HE Heckemann B Halfens RJ Lohrmann C. The Consolidated Framework for Implementation Research (CFIR): a useful theoretical framework for guiding and evaluating a guideline implementation process in a hospital-based nursing practice. BMC Nurs. (2015) 14:43. 10.1186/s12912-015-0088-4 26269693 PMC4533946

[7] Damschroder LJ Aron DC Keith RE Kirsh SR Alexander JA Lowery JC. Fostering implementation of health services research findings into practice: a consolidated framework for advancing implementation science. Implement Sci. (2009) 4:50. 10.1186/1748-5908-4-50 19664226 PMC2736161

[8] Shelton RC Lee M. Sustaining evidence-based interventions and policies: recent innovations and future directions in implementation science. Am J Public Health. (2019) 109:S132–S4. 10.2105/AJPH.2018.304913 30785794 PMC6383970

[9] Proctor E Silmere H Raghavan R Hovmand P Aarons G Bunger A. Outcomes for implementation research: conceptual distinctions, measurement challenges, and research agenda. Adm Policy Ment Health. (2011) 38:65–76. 10.1007/s10488-010-0319-7 20957426 PMC3068522

[10] Chambers DA Glasgow RE Stange KC. The dynamic sustainability framework: addressing the paradox of sustainment amid ongoing change. Implement Sci. (2013) 8:117. 10.1186/1748-5908-8-117 24088228 PMC3852739

[11] Luke DA Calhoun A Robichaux CB Elliott MB Moreland-Russell S. The program sustainability assessment tool: a new instrument for public health programs. Prev Chronic Dis. (2014) 11:130184. 10.5888/pcd11.130184 24456645 PMC3900326

[12] Proctor E Luke D Calhoun A McMillen C Brownson R McCrary S. Sustainability of evidence-based healthcare: research agenda, methodological advances, and infrastructure support. Implement Sci. (2015) 10:88. 10.1186/s13012-015-0274-5 26062907 PMC4494699

[13] Shelton RC Cooper BR Stirman SW. The sustainability of evidence-based interventions and practices in public health and health care. Annu Rev Public Health. (2018) 39:55–76. 10.1146/annurev-publhealth-040617-014731 29328872

[14] Bauer MS Damschroder L Hagedorn H Smith J Kilbourne AM. An introduction to implementation science for the non-specialist. BMC Psychol. (2015) 3:32. 10.1186/s40359-015-0089-9 26376626 PMC4573926

[15] Berwick DM. The science of improvement. JAMA. (2008) 299:1182–4. 10.1001/jama.299.10.1182 18334694

[16] Chassin MR Loeb JM. The ongoing quality improvement journey: next stop, high reliability. Health Aff (Millwood). (2011) 30:559–68. 10.1377/hlthaff.2011.0076 21471473

[17] Itri JN Bakow E Probyn L Kadom N Duong PT Gettle LM. The science of quality improvement. Acad Radiol. (2017) 24:253–62. 10.1016/j.acra.2016.05.010 28193375

[18] John Cantiello PK Shirley M Sabiheen A. The evolution of quality improvement in healthcare: Patient-centered care and health information technology applications. J Hospit Administr. (2016) 5:62–8. 10.5430/jha.v5n2p6235777803

[19] Marjoua Y Bozic KJ. Brief history of quality movement in US healthcare. Curr Rev Musculoskelet Med. (2012) 5:265–73. 10.1007/s12178-012-9137-8 22961204 PMC3702754

[20] Administration USDoHaHSHRaS. Quality Improvement. (2011). p. 1–19.

[21] Cayce J Savage T Hodge D Pickard K Myers P Powell K. Sustained reduction and prevention of neonatal and pediatric central line-associated bloodstream infection following a nurse-driven quality improvement initiative in a pediatric facility. J Assoc Vascul Access. (2018) 23:30–41. 10.1016/j.java.2017.11.002

[22] Linam WM Margolis PA Atherton H Connelly BL. Quality-improvement initiative sustains improvement in pediatric health care worker hand hygiene. Pediatrics. (2011) 128:e689–98. 10.1542/peds.2010-3587 21824885

[23] O'Donoghue SC DiLibero J Altman M. Leading sustainable quality improvement. Nurs Manage. (2021) 52:42–50. 10.1097/01.NUMA.0000724940.43792.86 33512883

[24] Lachman P Gondek D Edbrooke-Childs J Deighton J Stapley E. Perspectives of paediatric hospital staff on factors influencing the sustainability and spread of a safety quality improvement programme. BMJ Open. (2021) 11:e042163. 10.1136/bmjopen-2020-042163 33753434 PMC7986768

[25] National National Academies of Sciences E Medicine. Applying an implementation science approach to genomic medicine: workshop summary. Washington, DC: National Academies Press (2016). 27123510

[26] May C Finch T Mair F Ballini L Dowrick C Eccles M. Understanding the implementation of complex interventions in health care: the normalization process model. BMC Health Serv Res. (2007) 7:1–7. 10.1186/1472-6963-7-148 17880693 PMC2089069

[27] Scheirer MA Dearing JW. An agenda for research on the sustainability of public health programs. Am J Public Health. (2011) 101:2059–67. 10.2105/AJPH.2011.300193 21940916 PMC3222409

[28] Squires JE Graham ID Hutchinson AM Michie S Francis JJ Sales A. Identifying the domains of context important to implementation science: a study protocol. Implement Sci. (2015) 10:135. 10.1186/s13012-015-0325-y 26416206 PMC4584460

[29] Brownson RC Shelton RC Geng EH Glasgow RE. Revisiting concepts of evidence in implementation science. Implement Sci. (2022) 17:26. 10.1186/s13012-022-01201-y 35413917 PMC9004065

[30] Braithwaite J Ludlow K Testa L Herkes J Augustsson H Lamprell G. Built to last? the sustainability of healthcare system improvements, programmes and interventions: a systematic integrative review. BMJ Open. (2020) 10:e036453. 10.1136/bmjopen-2019-036453 32487579 PMC7265014

[31] Luke D Prewitt K Malone S. Understand Sustainability. (2020). Available online at: https://sustaintool.org/csat/understand/ (accessed July 25, 2022).

[32] Casimir G. Why children's hospitals are unique and so essential. Front Pediatr. (2019) 7:305. 10.3389/fped.2019.00305 31396498 PMC6664869

[33] Martinez-Castaldi C Silverstein M Bauchner H. Child vs. adult research: the gap in high-quality study design. Pediatrics. (2008) 122:52–7. 10.1542/peds.2007-2849 18595986

[34] Malone SM Seigel NS Newland JG Saito JM McKay VR. Understanding antibiotic prophylaxis prescribing in pediatric surgical specialties. Infect Control Hosp Epidemiol. (2020) 41:666–71. 10.1017/ice.2020.71 32252848 PMC8202117

[35] Malone S McKay VR Krucylak C Powell BJ Liu J Terrill C. A cluster randomized stepped-wedge trial to de-implement unnecessary post-operative antibiotics in children: the optimizing perioperative antibiotic in children (OPerAtiC) trial. Implement Sci. (2021) 16:29. 10.1186/s13012-021-01096-1 33741048 PMC7980649

[36] Wieczorek B Ascenzi J Kim Y Lenker H Potter C Shata NJ. PICU Up!: impact of a quality improvement intervention to promote early mobilization in critically ill children. Pediatr Crit Care Med. (2016) 17:e559–e66. 10.1097/PCC.0000000000000983 27759596 PMC5138131

[37] Leonard JC Josephson CD Luther JF Wisniewski SR Allen C Chiusolo F. Life-threatening bleeding in children: a prospective observational study. Crit Care Med. (2021) 49:1943–54. 10.1097/CCM.0000000000005075 33990098 PMC8516672

[38] Bratzler DW Dellinger EP Olsen KM Perl TM Auwaerter PG Bolon MK. Clinical practice guidelines for antimicrobial prophylaxis in surgery. Am J Health Syst Pharm. (2013) 70:195–283. 10.2146/ajhp120568 23327981

[39] Marra A Ely EW Pandharipande PP Patel MB. The ABCDEF bundle in critical care. Crit Care Clin. (2017) 33:225–43. 10.1016/j.ccc.2016.12.005 28284292 PMC5351776

[40] Consunji R Elseed A El-Menyar A Sathian B Rizoli S Al-Thani H. The effect of massive transfusion protocol implementation on the survival of trauma patients: a systematic review and meta-analysis. Blood Transfus. (2020) 18:434–45. 32955420 10.2450/2020.0065-20 PMC7605882

[41] Malone S Prewitt K Hackett R Lin JC McKay V Walsh-Bailey C. The Clinical Sustainability Assessment tool: measuring organizational capacity to promote sustainability in healthcare. Implement Sci Commun. (2021) 2:77. 10.1186/s43058-021-00181-2 34274004 PMC8285819

[42] Agulnik A Malone S Puerto-Torres M Gonzalez-Ruiz A Vedaraju Y Wang H. Reliability and validity of a Spanish-language measure assessing clinical capacity to sustain Paediatric Early Warning Systems (PEWS) in resource-limited hospitals. BMJ Open. (2021) 11:e053116. 10.1136/bmjopen-2021-053116 34670767 PMC8529978

[43] Solberg LI Asche SE Margolis KL Whitebird RR. Measuring an organization's ability to manage change: the change process capability questionnaire and its use for improving depression care. Am J Med Qual. (2008) 23:193–200. 10.1177/1062860608314942 18539980

[44] Bland JM Altman DG. Statistics notes: Cronbach's alpha. Bmj. (1997) 314:572. 10.1136/bmj.314.7080.572 9055718 PMC2126061

[45] EvidenceNOW: Change Process Capability Questionnaire (CPCQ) Scoring Guidance Rockville MD: Agency for Healthcare Research and Quality; [updated February (2019). Available from: https://www.ahrq.gov/evidencenow/results/research/cpcq-scoring.html

[46] Luke DA. Multilevel Modeling. 2nd ed. Entwisle B, editor. Thousand Oaks, California: SAGE Publications (2020). p. 107.

[47] Schwartz SP Rehder KJ. Quality improvement in pediatrics: past, present, and future. Pediatr Res. (2017) 81:156–61. 10.1038/pr.2016.192 27673419

[48] Lengnick-Hall R Stadnick NA Dickson KS Moullin JC Aarons GA. Forms and functions of bridging factors: specifying the dynamic links between outer and inner contexts during implementation and sustainment. Implement Sci. (2021) 16:34. 10.1186/s13012-021-01099-y 33794956 PMC8015179

[49] Easterling D Perry AC Woodside R Patel T Gesell SB. Clarifying the concept of a learning health system for healthcare delivery organizations: implications from a qualitative analysis of the scientific literature. Learn Health Syst. (2021) 6. 10.1002/lrh2.10287 35434353 PMC9006535

[50] Moullin JC Sklar M Green A Dickson KS Stadnick NA Reeder K. Advancing the pragmatic measurement of sustainment: a narrative review of measures. Implement Sci Commun. (2020) 1:76. 10.1186/s43058-020-00068-8 32964208 PMC7499830

[51] Flanagan ME Ramanujam R Doebbeling BN. The effect of provider- and workflow-focused strategies for guideline implementation on provider acceptance. Implement Sci. (2009) 4:71. 10.1186/1748-5908-4-71 19874607 PMC2777118

[52] Scheirer MA. Linking sustainability research to intervention types. Am J Public Health. (2013) 103:e73–80. 10.2105/AJPH.2012.300976 23409904 PMC3673273

[53] Batalden PB Davidoff F. What is “quality improvement” and how can it transform healthcare? Qual Saf Health Care. (2007) 16:2–3. 10.1136/qshc.2006.022046 17301192 PMC2464920

[54] Mittman BS. 19 Implementation Science in Health Care. Dissemination and implementation research in health: translating science to practice. Jama. (2012) 2012:1400. 10.1093/acprof:oso/9780199751877.003.0019

[55] Wensing M. Implementation science in healthcare: Introduction and perspective. Z Evid Fortbild Qual Gesundhwes. (2015) 109:97–102. 10.1016/j.zefq.2015.02.014 26028446

[56] Rubenstein LV Pugh J. Strategies for promoting organizational and practice change by advancing implementation research. J Gen Intern Med. (2006) 21:S58. 10.1007/s11606-006-0276-8 16637962 PMC2557137

