# Supplementary material for: Sustainability in pediatric hospitals: An exploration at the intersection of quality improvement and implementation science
Source: Front Health Serv. 2022 Nov 10;2:1005802. doi: 10.3389/frhs.2022.1005802 (PMC10012775; doi:10.3389/frhs.2022.1005802)
Supplement: Supplementary file 1 [file Data_Sheet_1.docx]

**Clinical Sustainability Assessment Tool**

# **What is clinical sustainability capacity?**

# We define clinical sustainability capacity as the ability of an organization to maintain structured clinical care practices over time and to evolve and adapt these practices in response to new information.

# **Why is clinical sustainability capacity important?**

Without sustaining effective practices over time, we risk not being able to see the full return on our nation’s investment in clinical and translational science. Successful implementation of new practices in clinical or healthcare settings is affected by a number of organizational, financial, regulatory, and political factors. To maintain these benefits, clinical settings and healthcare organizations must support these clinical practices in a number of ways. With knowledge of these critical factors, stakeholders can build capacity for sustainability of a clinical practice and position their efforts for long term success.

# **What is the purpose of this tool?**

This tool will help structure an assessment of your group's current capacity for sustainability across a range of specific organizational and contextual factors. Your responses will identify sustainability strengths and challenges. You can then use results to guide sustainability action planning for your clinical practice.

# **Helpful definitions**

The Clinical Sustainability Assessment Tool can be used in a wide variety of clinical practice settings. Before starting the assessment, you should identify the specific clinical practice you will be assessing.

Below are a few definitions of terms that are frequently used throughout the tool.

- **Practice** refers to the set of formal organized activities that you want to sustain over time. Such activities could occur in a variety of clinical settings.
- **Organization** encompasses all the parent organizations or agencies in which the practice is housed. Depending on the practice, the organization may refer to a health center, a hospital, etc.
- **Community** refers to the stakeholders who may benefit from or who may guide the practice. This could include clinical staff, leadership, care recipients and their families, etc. Community does not refer to a specific town or neighborhood.

The next question is to serve as a reference point for the following survey. Some examples of clinical practices are: antibiotic stewardship, a new surgical procedure, electronic medical record order sets, or hand hygiene.

**The name of the practice or set of activities I am assessing is:**

In the following questions, you will rate your practice across a range of specific factors that affect sustainability. Please respond to as many items as possible. If you truly feel you are not able to answer an item, you may select “NA.” **For each statement, circle the number that best indicates the extent to which your practice has or does the following things.**

**Engaged Staff & Leadership:** Having supportive frontline staff and management within the organization

|  | **To little**  **or no extent** | | | | **To a very**  **great extent** | | | | **Not able to answer** |
| --- | --- | --- | --- | --- | --- | --- | --- | --- | --- |
| 1. The practice engages leadership and staff throughout the process. | **1** | **2** | **3** | **4** | | **5** | **6** | **7** | **NA** |
| 1. Clinical champions of the practice are recognized and respected. | **1** | **2** | **3** | **4** | | **5** | **6** | **7** | **NA** |
| 1. The practice has engaged, ongoing champions. | **1** | **2** | **3** | **4** | | **5** | **6** | **7** | **NA** |
| 1. The practice has a leadership team made of multiprofessional partnerships. | **1** | **2** | **3** | **4** | | **5** | **6** | **7** | **NA** |
| 1. The practice has team-based collaboration and infrastructure. | **1** | **2** | **3** | **4** | | **5** | **6** | **7** | **NA** |

**For each statement, circle the number that best indicates the extent to which your practice has or does the following things.**

**Engaged Stakeholders:** Having external support and engagement for the practice.

|  | **To little**  **or no extent** | | | | **To a very**  **great extent** | | | | **Not able to answer** |
| --- | --- | --- | --- | --- | --- | --- | --- | --- | --- |
| 1. The practice engages the patient and family members as stakeholders. | **1** | **2** | **3** | **4** | | **5** | **6** | **7** | **NA** |
| 1. There is respect for all stakeholders involved in the practice. | **1** | **2** | **3** | **4** | | **5** | **6** | **7** | **NA** |
| 1. The practice is valued by a diverse set of stakeholders. | **1** | **2** | **3** | **4** | | **5** | **6** | **7** | **NA** |
| 1. The practice engages other medical teams and community partnerships as appropriate. | **1** | **2** | **3** | **4** | | **5** | **6** | **7** | **NA** |
| 1. The practice team has the ability to respond to stakeholder feedback about the practice. | **1** | **2** | **3** | **4** | | **5** | **6** | **7** | **NA** |

**Organizational Readiness:** Having the internal support and resources needed to effectively manage the practice

|  | **To little**  **or no extent** | | | | **To a very**  **great extent** | | | | **Not able to answer** |
| --- | --- | --- | --- | --- | --- | --- | --- | --- | --- |
| 1. Organizational systems are in place to support the various practice needs. | **1** | **2** | **3** | **4** | | **5** | **6** | **7** | **NA** |
| 1. The practice fits in well with the culture of the team. | **1** | **2** | **3** | **4** | | **5** | **6** | **7** | **NA** |
| 1. The practice has feasible and sufficient resources (e.g., time, space, funding) to achieve its goals. | **1** | **2** | **3** | **4** | | **5** | **6** | **7** | **NA** |
| 1. The practice has adequate staff to achieve its goals. | **1** | **2** | **3** | **4** | | **5** | **6** | **7** | **NA** |
| 1. The practice is well integrated into the operations of the organization | **1** | **2** | **3** | **4** | | **5** | **6** | **7** | **NA** |

**For each statement, circle the number that best indicates the extent to which your practice has or does the following things.**

**Workflow Integration:** Designing the practice to fit into existing practices and technologies

|  | **To little**  **or no extent** | | | | **To a very**  **great extent** | | | | **Not able to answer** |
| --- | --- | --- | --- | --- | --- | --- | --- | --- | --- |
| 1. The practice is built into the clinical workflow. | **1** | **2** | **3** | **4** | | **5** | **6** | **7** | **NA** |
| 1. The practice is easy for clinicians to use. | **1** | **2** | **3** | **4** | | **5** | **6** | **7** | **NA** |
| 1. The practice integrates well with established clinical practices. | **1** | **2** | **3** | **4** | | **5** | **6** | **7** | **NA** |
| 1. The practice aligns well with other clinical systems (e.g., EMR). | **1** | **2** | **3** | **4** | | **5** | **6** | **7** | **NA** |
| 1. The practice is designed to be used consistently. | **1** | **2** | **3** | **4** | | **5** | **6** | **7** | **NA** |

**Implementation & Training:** Using processes that guide the direction, goals and strategies of the practice

|  | **To little**  **or no extent** | | | | **To a very**  **great extent** | | | | **Not able to answer** |
| --- | --- | --- | --- | --- | --- | --- | --- | --- | --- |
| 1. The practice clearly outlines roles and responsibilities for all staff. | **1** | **2** | **3** | **4** | | **5** | **6** | **7** | **NA** |
| 1. The reason for the practice is clearly communicated to and understood by all staff. | **1** | **2** | **3** | **4** | | **5** | **6** | **7** | **NA** |
| 1. Staff receive ongoing coaching, feedback, and training. | **1** | **2** | **3** | **4** | | **5** | **6** | **7** | **NA** |
| 1. Practice implementation is guided by feedback from stakeholders. | **1** | **2** | **3** | **4** | | **5** | **6** | **7** | **NA** |
| 1. The practice has ongoing education across professions. | **1** | **2** | **3** | **4** | | **5** | **6** | **7** | **NA** |

**For each statement, circle the number that best indicates the extent to which your practice has or does the following things.**

**Monitoring & Evaluation:** Assessing the practice to inform planning and document results

|  | **To little**  **or no extent** | | | | **To a very**  **great extent** | | | | **Not able to answer** |
| --- | --- | --- | --- | --- | --- | --- | --- | --- | --- |
| 1. The practice has measurable process components, outcomes, and metrics. | **1** | **2** | **3** | **4** | | **5** | **6** | **7** | **NA** |
| 1. Evaluation and monitoring of the practice are reviewed on a consistent basis. | **1** | **2** | **3** | **4** | | **5** | **6** | **7** | **NA** |
| 1. The practice has clear documentation to guide process and outcome evaluation. | **1** | **2** | **3** | **4** | | **5** | **6** | **7** | **NA** |
| 1. Practice monitoring, evaluation, and outcomes data are routinely reported to the clinical care team. | **1** | **2** | **3** | **4** | | **5** | **6** | **7** | **NA** |
| 1. The practice process components, outcomes, and metrics are easily assessed and audited. | **1** | **2** | **3** | **4** | | **5** | **6** | **7** | **NA** |

**Outcomes & Effectiveness:** Understanding and measuring practice outcomes and impact

|  | **To little**  **or no extent** | | | | **To a very**  **great extent** | | | | **Not able to answer** |
| --- | --- | --- | --- | --- | --- | --- | --- | --- | --- |
| 1. The practice has evidence of beneficial outcomes. | **1** | **2** | **3** | **4** | | **5** | **6** | **7** | **NA** |
| 1. The practice is associated with improvement in patient outcomes that are clinically meaningful. | **1** | **2** | **3** | **4** | | **5** | **6** | **7** | **NA** |
| 1. The practice is clearly linked to positive health or clinical outcomes. | **1** | **2** | **3** | **4** | | **5** | **6** | **7** | **NA** |
| 1. The practice is cost-effective. | **1** | **2** | **3** | **4** | | **5** | **6** | **7** | **NA** |
| 1. The practice has clear advantages over alternatives. | **1** | **2** | **3** | **4** | | **5** | **6** | **7** | **NA** |


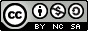
The *Clinical Sustainability Assessment Tool* is a copyrighted instrument of Washington University, St Louis MO. All rights reserved. This work is licensed under a [Creative Commons Attribution-NonCommercial- ShareAlike License](http://creativecommons.org/licenses/by-nc/3.0/). If you modify this tool, please notify the Center for Public Health Systems Science. By using the *Clinical Sustainability Assessment Tool* you understand and agree to these terms of use and agree that Washington University bears no responsibility to you or any third party for the consequences of your use of the tool. If you would like more information about how to use this tool with your program or would like to learn about our sustainability workshops and webinars, visit <http://www.sustaintool.org>. August 2019

**Intervention**

**The following questions will ask about the clinical practice or set of activities you are assessing.**

1. Please rate the strength of the scientific evidence supporting your clinical practice.
2. Very weak
3. Weak
4. Neither weak nor strong
5. Strong
6. Very strong
7. Don’t know/NA
8. Approximately how long has the practice been implemented at your workplace?
9. Less than 1 year
10. 1 – 2 years
11. 3 – 5 years
12. 6-10 years
13. Greater than 10 years
14. Don’t know
15. How important is the clinical practice to provide quality care to your patient population?
    1. Not at all important
    2. Somewhat unimportant
    3. Neither important nor unimportant
    4. Somewhat important
    5. Very important
16. How achievable was or is this clinical practice to implement within your organization?
    1. Very difficult
    2. Somewhat difficult
    3. Neither easy nor difficult
    4. Somewhat easy
    5. Very easy
17. Regarding patients under my care, they can expect to receive this intervention
    1. None of the time
    2. Some of the time
    3. Most of the time
    4. All of the time

**Organization**

**The following questions will ask about your organization and environment in which you work.**

1. To the best of your knowledge, how many clinical staff are employed in your organization?
   1. less than 50
   2. 50 – 249
   3. 250 +
2. Which of the following best describes your clinical work environment?
   1. Academic hospital
   2. Community hospital
   3. Private hospital
   4. Community Health Center
   5. Ambulatory care
   6. Urgent care
   7. Clinic (private practice)
   8. Nursing home
   9. Other (please specify): ___________________________________

8. Which of the following best describes your place of work?

1. Urban
2. Suburban
3. Rural

**Please indicate how much you agree or disagree with each of the following statements.**

|  | Strongly Disagree | Somewhat Disagree | Neither agree nor disagree | Somewhat agree | Strongly Agree |
| --- | --- | --- | --- | --- | --- |
| 1. Our resources (personnel, time, financial) are too tightly limited to improve care quality now. | 1 | 2 | 3 | 4 | 5 |
| 1. Our clinical team understands and uses quality improvement skills effectively. | 1 | 2 | 3 | 4 | 5 |
| 1. Our clinical team has changed or created systems in the organization that make it easier to provide high quality care. | 1 | 2 | 3 | 4 | 5 |
| 1. We choose new processes of care that are more advantageous than the old to everyone involved (patients, clinicians, and our entire clinical team). | 1 | 2 | 3 | 4 | 5 |
| 1. The working environment in our clinical team is collaborative and cohesive, with shared sense of purpose, cooperation, and willingness to contribute to the common good. | 1 | 2 | 3 | 4 | 5 |
| 1. Our clinical team has greatly improved quality of care in the past year. | 1 | 2 | 3 | 4 | 5 |

**Participant**

**The following questions will ask about your work. Please indicate your response for each question or statement.**

1. What is your primary service setting?
   1. Inpatient
   2. Outpatient
   3. Both inpatient and outpatient
   4. N/A – Does not apply to my work
2. What population do you primarily work with?
   1. Adult
   2. Pediatrics
   3. Both adult and Pediatrics
   4. N/A – Does not apply to my work
3. What is your primary profession?
4. Advanced Practice Provider/Nurse Practitioner
5. Behavioral Health
6. Healthcare Administration
7. Nurse
8. Oral health
9. Pharmacist
10. Physician
11. Psychologist
12. Public health
13. Rehabilitation specialist: physical/occupational therapist
14. Rehabilitation specialist: speech/music/art/child life/education
15. Researcher
16. Respiratory Therapist
17. Social work
18. Other (please list): ___________________________________
19. What is your primary current position?
20. Bedside provider/ direct patient care
21. Leadership/management
22. Administrative
23. Research
24. Other (please specify): _____________________________________
25. In relation to the clinical practice, what is your primary role?
26. Leading (e.g., point person or champion)
27. Administration
28. Participating
29. Evaluator
30. Clinical staff
31. Other _____________________________
